# Effects of Long-Term Supplementation with *Centella asiatica* (L.) Urb. Extract (CA-HE50) on Macular Pigment Optical Density: A Randomized, Double-Blind, Placebo-Controlled Trial

**DOI:** 10.3390/nu18060905

**Published:** 2026-03-12

**Authors:** Hyang-Im Baek, Inhye Kim, Jaewoo Bae, Jeong Eun Kwon, Se-Chan Kang

**Affiliations:** 1 Department of Food Science & Nutrition, Woosuk University, Wanju 55338, Republic of Korea; 2 GENENCELL Co., Ltd., Yongin 16950, Republic of Korea; inhyekim@genencell.co.kr; 3 3H LABS Research Institute, 3H LABS Co., Ltd., Goyang 10391, Republic of Korea; jerry@3h-labs.com; 4 BioMedical Research Institute, Kyung Hee University, Yongin 17104, Republic of Korea; jjung1169@hanmail.net; 5 Department of Oriental Medicine Biotechnology, College of Life Sciences, Kyung Hee University, Yongin 17104, Republic of Korea

**Keywords:** *Centella asiatica*, asiaticoside, macular pigment optical density, age-related macular degeneration, eye health, functional food

## Abstract

**Background/Objectives**: Macular pigment optical density (MPOD) is a nutrition-responsive biomarker that indicates the antioxidant status of the macula. This study aimed to evaluate the effects of long-term supplementation with a standardized *Centella asiatica* (L.) Urb. extract (CA-HE50) on MPOD in a randomized, double-blind, placebo-controlled clinical trial. **Methods**: Eighty men and women aged 45–65 years, with baseline MPOD values between 0.2 and 0.4, were randomly assigned to receive either CA-HE50 (300 mg/day, *n* = 40) or a placebo (*n* = 40) for 6 months. Efficacy was assessed by measuring MPOD at baseline and on Days 60, 120, and 180. The primary efficacy endpoint was the change in MPOD from baseline to Day 180. Safety was evaluated through monitoring adverse events, vital signs, and clinical laboratory tests. **Results**: By Day 180, supplementation with CA-HE50 resulted in a statistically significant increase in MPOD compared to the placebo in the right eye, left eye, and the average of both eyes (all *p* < 0.001). Significant between-group differences were also observed at Day 120, indicating a time-dependent improvement in MPOD. Additionally, the proportion of responders was significantly higher in the CA-HE50 group compared to the placebo group (*p* < 0.001). CA-HE50 was well tolerated, with no serious adverse events or clinically relevant safety concerns identified during the intervention period. **Conclusions**: Long-term supplementation with *C. asiatica* extract significantly improved MPOD, supporting its potential role in enhancing macular nutritional status. These findings suggest that CA-HE50 may serve as a beneficial dietary intervention for maintaining macular health.

## 1. Introduction

Ageing is a major factor in the progressive vulnerability of the retina and the degenerative changes seen in the macula. This vulnerability is a result of cumulative oxidative stress, declining antioxidant defenses, and the gradual deterioration of retinal pigment epithelial (RPE) cells over time [1,2]. However, emerging evidence suggests that macular vulnerability is not limited to older adults. Lifelong exposure to light and modern lifestyle factors imposes oxidative and photo-oxidative stress on retinal tissues, leading to earlier and more widespread susceptibility of the macula throughout adulthood [3,4]. Consequently, midlife is a crucial period where nutritional strategies can effectively support macular resilience before irreversible structural damage occurs, especially for adults at higher risk of macular vulnerability.

The retina is particularly vulnerable to oxidative stress due to its high metabolic demand, intense oxygen consumption, ongoing exposure to light, and high levels of polyunsaturated fatty acids [5]. Prolonged oxidative stress disrupts retinal homeostasis by impairing RPE cell function and photoreceptor integrity, thus increasing retinal vulnerability in both older and younger populations [2]. These factors highlight the need for long-term nutritional interventions that enhance the body’s antioxidant capacity and cellular defense mechanisms, rather than relying on short-term symptomatic treatments.

Macular pigment optical density (MPOD) is a non-invasive biomarker that reflects the antioxidant status of the macula, a specialized region of the retina responsible for central and high-acuity vision [6,7,8]. The macular pigment, primarily composed of the dietary carotenoids lutein, zeaxanthin, and meso-zeaxanthin, serves to filter harmful light and scavenge reactive oxygen species, thus protecting retinal tissues from photo-oxidative damage [9,10,11]. Significant variability in MPOD levels has been observed among healthy adults, including those in middle age, with lower MPOD levels indicating a greater susceptibility of the macula to oxidative stress [7]. Furthermore, reduced MPOD levels have consistently been found in individuals with age-related macular degeneration (AMD), supporting the concept that MPOD represents a modifiable intermediate marker linking cumulative oxidative stress to long-term macular health outcomes rather than a consequence of overt retinal disease [7,12,13].

Nutritional interventions aimed at MPOD have primarily focused on increasing dietary intake or supplementation of lutein and zeaxanthin [14,15]. Randomized controlled trials have shown that carotenoid supplementation can increase MPOD in healthy adults [16,17]. However, significant variability among individuals and plateauing effects during prolonged supplementation have been consistently observed [18,19,20,21]. Factors such as absorption efficiency, plasma transport, tissue distribution, baseline MPOD, and the retinal oxidative environment all play a role in macular pigment accumulation [20,22]. These limitations indicate that macular pigment levels may be influenced by retinal cellular conditions beyond just carotenoid availability, underscoring the need for complementary nutritional strategies that enhance the retinal antioxidant environment.

*Centella asiatica* (L.) Urb., commonly known as Gotu Kola or Indian pennywort, is a herbaceous perennial plant in the Apiaceae (Umbelliferae) family. It has been traditionally utilized in South and Southeast Asia for its nutritional and therapeutic benefits [23,24,25]. *C. asiatica* is well-regarded for its antimicrobial, antioxidant, anti-inflammatory, neuroprotective, cytoprotective, and wound-healing properties, which have sparked growing interest in its use in contemporary nutritional and clinical research [26,27]. The major bioactive compounds in *C. asiatica* extract are triterpene glycosides (saponins), including asiaticoside and madecassoside, along with their aglycones (sapogenins), such as asiatic acid and madecassic acid [28,29]. These triterpenoid compounds have been found to influence oxidative stress responses, inflammatory signaling, and cellular defense mechanisms, offering a mechanistic foundation for the cytoprotective and antioxidant effects attributed to *C. asiatica* extracts [30,31].

In this study, we utilized the standardized *C. asiatica* extract (CA-HE50) that has previously shown protective effects on human retinal pigment epithelial (RPE) cells in preclinical investigations [32,33]. These studies demonstrated that CA-HE50 safeguards RPE cells from oxidative and photo-oxidative stress by activating endogenous antioxidant pathways, including nuclear factor erythroid 2-related factor 2 (Nrf2) and heme oxygenase-1 (HO-1), while also suppressing apoptosis-related signaling. Furthermore, oral administration of CA-HE50 has been shown to maintain retinal structure and visual function-related protein expression in animal models of chemically induced retinal degeneration, supporting its role in enhancing retinal resilience under oxidative stress.

Importantly, CA-HE50 does not contain macular carotenoids, meaning it does not directly contribute to the formation of macular pigment. This distinction sets CA-HE50 apart from traditional MPOD-targeted nutritional interventions and offers a unique framework to assess whether changes in the retinal antioxidant environment alone can influence MPOD levels. Since MPOD reflects not only the availability of carotenoids but also the overall oxidative environment of the macula, any changes in MPOD following CA-HE50 supplementation may indicate improved retinal resilience rather than direct pigment delivery.

Although there is growing nonclinical evidence supporting the retinal protective effects of CA-HE50, its impact on MPOD has yet to be evaluated in clinical settings. We hypothesized that long-term supplementation with CA-HE50 would enhance MPOD by improving the retinal antioxidant environment, independent of direct carotenoid supply. Therefore, this study aimed to investigate whether 6-month supplementation with CA-HE50 could improve MPOD in adults aged 45–65 years with low baseline MPOD.

## 2. Materials and Methods

### 2.1. Ethics Statement

This clinical trial was conducted following the ethical principles outlined in the Declaration of Helsinki, the Code of Federal Regulations (CFR), and the International Council for Harmonisation guidelines for Good Clinical Practice (ICH-GCP). The Institutional Review Board (IRB) of Narayana Nethralaya in Bangalore, India, reviewed and approved the study protocol, informed consent form, and all relevant documents (IRB approval No.: C/2019/05/01; approval date: 16 May 2019) prior to the initiation of the study.

The trial was prospectively registered with the Clinical Trials Registry India (CTRI Number: CTRI/2019/05/019392; registered on 28 May 2019). Written informed consent was obtained from all participants before enrollment, following a thorough explanation of the study’s objectives, procedures, potential risks, and anticipated benefits. Participants were informed of their right to withdraw from the study at any time without facing any consequences.

The study was conducted and reported in accordance with the Consolidated Standards of Reporting Trials (CONSORT) guidelines for randomized controlled trials.

### 2.2. Study Participants

Participants were recruited from Narayana Nethralaya in Bangalore, India, during the study’s recruitment period. All potential participants underwent screening according to the predefined study protocol and were enrolled after providing written informed consent.

Eligible men and women aged 45 to 65 years had to have baseline MPOD values between 0.2 and 0.4, as measured by heterochromatic flicker photometry. Participants were required to have no clinically significant ocular conditions, though those with clinically non-significant accommodation-related visual problems could participate at the investigator’s discretion. Additional inclusion criteria included the ability to follow the dietary regimen outlined in the study protocol, willingness to attend regular follow-up visits throughout the intervention period, and the capability to provide written informed consent before any study-related procedures.

Exclusion criteria included a history of hypersensitivity to herbal extracts or dietary supplements, as well as the presence of ocular diseases such as glaucoma, advanced cataracts, pan-retinal degeneration, or other clinically significant retinal disorders. Systemic conditions that could influence study outcomes, including hypercholesterolemia, renal or hepatic disorders, diabetes mellitus, or other debilitating diseases, were also grounds for exclusion. Participants currently undergoing treatment with herbal preparations or allopathic ocular medications or receiving other concomitant medications that could potentially influence the study outcomes (e.g., sedatives or other centrally acting agents) were not eligible. Additional criteria excluded individuals who had participated in another investigational drug study or clinical trial within the previous three months, as well as anyone deemed unsuitable for inclusion by the investigator.

A total of 80 eligible participants were randomized to receive either the CA-HE50 supplement or a placebo, following the predefined study protocol. Screening, enrollment, randomization, and follow-up of participants were carried out according to the study procedures, with the flow of participants through each stage of the trial presented in the CONSORT flow diagram.

### 2.3. Study Design

This study was designed as a single-center, randomized, double-blind, placebo-controlled, parallel-group clinical trial conducted at Narayana Nethralaya in Bangalore, India, from May to December 2019.

**Figure 1:**
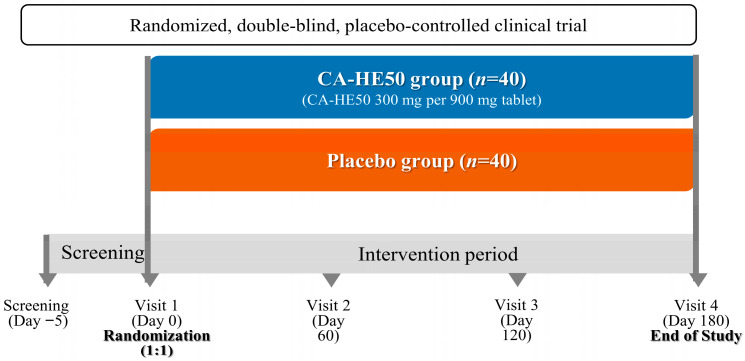
Study design and intervention schedule.

The trial included a screening visit, a baseline visit (Visit 1, Day 0), and three scheduled follow-up visits throughout the intervention period. Baseline assessments were conducted during the screening visit and Visit 1 (Day 0) prior to randomization. A total of 80 eligible participants were randomly assigned in a 1:1 ratio to receive either CA-HE50 (*n* = 40) or a placebo (*n* = 40), using a computer-generated randomization sequence created with SAS software (version 9.4) based on a simple randomization method. The allocation sequence was implemented according to a predefined randomization schedule, and both participants and investigators were blinded to the treatment assignment throughout the study. The intervention period lasted six months, during which participants attended three follow-up visits at Visit 2 (Day 60), Visit 3 (Day 120), and Visit 4 (Day 180). At each follow-up visit, study procedures were carried out according to the study protocol, including assessments of intervention compliance, safety monitoring, and evaluation of efficacy outcomes. The overall study design and intervention schedule are illustrated in Figure 1.

Both participants and all study personnel involved in participant management, outcome assessment, and data analysis remained blinded to treatment allocation throughout the duration of the study. To minimize potential confounding effects, participants were instructed to maintain their usual dietary habits and lifestyle patterns during the trial.

**Figure 2:**
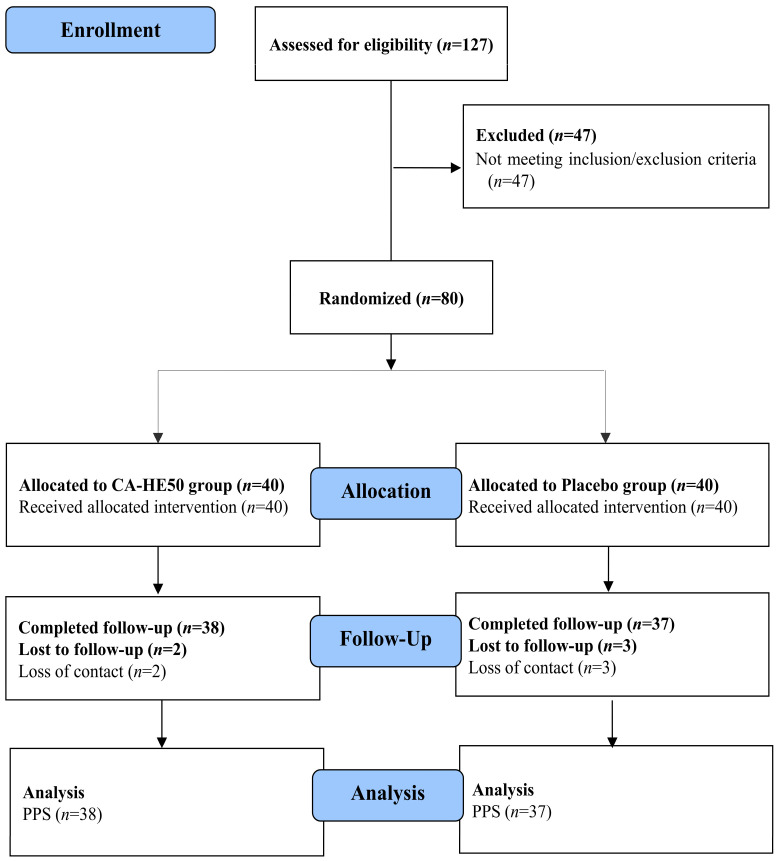
Flow diagram of participant enrollment, randomization, follow-up, and analysis.

The flow of participants through the study, including screening, randomization, follow-up, and analysis, is presented in Figure 2.

### 2.4. Study Products and Interventions

The study product used in this research was a standardized extract of *C. asiatica* (CA-HE50), provided by GENENCELL Co., Ltd. (Yongin, Republic of Korea). The manufacturing process of CA-HE50 has been detailed in previous studies [32,33] and follows a reproducible and standardized procedure to ensure consistency and quality. In summary, dried *C. asiatica* was extracted with 50% ethanol for 8 h at 80 °C, then concentrated and spray-dried to produce the standardized powdered extract (CA-HE50).

**Figure 3:**
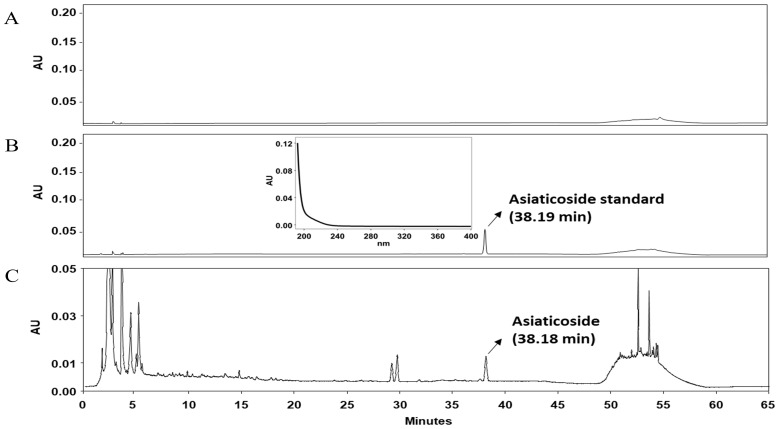
Representative HPLC chromatograms of (**A**) blank, (**B**) asiaticoside standard, and (**C**) *Centella asiatica* extract (CA-HE50), showing the characteristic peak of asiaticoside used for extract standardization.

This extract is standardized based on its major triterpenoid constituents, with asiaticoside quantified and controlled at 14.1 mg/g to maintain batch-to-batch uniformity of the active compound. The asiaticoside content was determined by high-performance liquid chromatography (HPLC) using a C18 column with UV detection at 206 nm. Representative chromatograms of the blank, asiaticoside standard, and CA-HE50 extract are presented in Figure 3.

Participants in the intervention group took one tablet daily containing 300 mg of CA-HE50, administered orally with water for 6 months. The detailed composition of the active and placebo tablets, including excipients, is summarized in Table 1. The placebo tablets, made primarily of microcrystalline cellulose, contained no *C. asiatica* extract or other bioactive components. To ensure randomization and blinding, both the CA-HE50 and placebo tablets were identical in appearance, weight, taste, and packaging and were provided in the same dosage form. The placebo formulation contained the same excipients as the active tablet but without the CA-HE50. Both participants and all study personnel involved in participant management, outcome assessment, and data analysis remained blinded to treatment allocation throughout the study period.

Compliance with the study intervention was evaluated at each scheduled visit through participant interviews and tablet accountability. Participants were instructed to take the assigned tablet around the same time each day. Additionally, the use of other herbal products, nutritional supplements, or medications known to impact retinal or macular health was restricted during the intervention period.

### 2.5. Efficacy Outcome Measures

Efficacy assessments were conducted at baseline and each scheduled follow-up visit during the intervention period, utilizing consistent measurement equipment throughout the study to ensure reliability.

The primary efficacy variable chosen for this study was MPOD, a non-invasive biomarker that reflects the antioxidant status of the macula. MPOD indicates the accumulation of protective carotenoids in the macular region and is commonly used to evaluate macular vulnerability to oxidative and photo-oxidative stress. Measurements of MPOD were taken in the right eye, left eye, and as an average of both eyes using a validated, non-invasive technique based on heterochromatic flicker photometry, which is frequently employed in clinical and nutritional intervention studies. All measurements were performed under standardized testing conditions by trained personnel.

MPOD assessments were performed at baseline and at Visit 2 (Day 60), Visit 3 (Day 120), and Visit 4 (Day 180). This approach allowed for the characterization of longitudinal changes throughout the intervention period.

The primary efficacy outcome was the change in MPOD from baseline to Day 180, which indicated the effect of 6-month supplementation with CA-HE50 on macular pigment status. Secondary efficacy outcomes included changes in MPOD at intermediate time points (Days 60 and 120), comparisons of MPOD changes between groups at each follow-up visit, and an analysis of MPOD responses in the right eye, left eye, and the average of both eyes. This evaluation aimed to assess the temporal pattern and bilateral consistency of the intervention’s effects. In addition, a responder analysis was performed to explore individual variability in MPOD responses. Responders were defined as participants who showed an increase in MPOD from baseline to Day 180, indicating a directional improvement in macular pigment during the intervention period.

### 2.6. Safety Outcome Measures

Safety assessments involved evaluating the incidence of adverse events (AEs), vital signs, and clinical laboratory parameters to determine the tolerability of CA-HE50 supplementation.

AEs were continuously monitored throughout the study and documented at each scheduled visit based on participant self-reports and investigator evaluations. All reported AEs were assessed for severity, duration, and potential relationship to the study product.

Vital signs—including systolic blood pressure (SBP), diastolic blood pressure (DBP), pulse rate, heart rate, respiratory rate, and body temperature—were measured under standardized conditions.

Clinical laboratory assessments were conducted at baseline and again on Day 180. Hematological parameters evaluated included hemoglobin, white blood cell count (WBC), differential leukocyte counts (neutrophils, lymphocytes, monocytes, and eosinophils), red blood cell count (RBC), platelet count, packed cell volume (PCV), mean corpuscular volume (MCV), mean corpuscular hemoglobin (MCH), and mean corpuscular hemoglobin concentration (MCHC). Blood biochemistry analyses assessed renal function markers (creatinine and blood urea), liver function markers [total bilirubin, aspartate aminotransferase (AST), alanine aminotransferase (ALT), and alkaline phosphatase (ALP)], serum proteins, and electrolytes (sodium, potassium, and chloride). All tests were performed after a 12 h overnight fast.

### 2.7. Statistical Analysis

This study aimed to assess the impact of 6-month supplementation with CA-HE50 on MPOD in comparison to a placebo. The sample size was calculated based on the primary efficacy endpoint, referencing previous clinical studies that utilized a similar design and focused on MPOD-related outcomes [34]. With a two-sided significance level set at 5% and a statistical power of 80%, the estimated sample size was 36 participants per group. To account for a potential dropout rate of 10%, a total of 80 participants (40 per group) were scheduled for randomization.

All statistical analyses were performed using SAS software (version 9.4; SAS Institute Inc., Cary, NC, USA). Continuous variables are reported as mean ± standard deviation (SD), while categorical variables are presented as number (percentage).

Efficacy analyses primarily utilized the per-protocol (PP) analysis set, which included participants who completed the study without major protocol deviations, adhered adequately to the study product, and had post-baseline measurements for the primary efficacy outcome. To enhance the robustness and consistency of the findings, additional analyses were conducted using the intention-to-treat (ITT) analysis set, which are also presented for reference. ITT analyses included all randomized participants, and missing data were not imputed.

Within-group comparisons of changes from baseline were performed using the paired *t*-test, while between-group comparisons were assessed with the independent *t*-test. Categorical variables were compared between groups using either the chi-square test or Fisher’s exact test, as appropriate. In addition, longitudinal changes in MPOD over time were evaluated using a linear mixed-effects model including group, time, and group × time interaction to account for repeated measurements within participants. Effect sizes for between-group differences were calculated using Cohen’s d.

All statistical tests were two-sided, and a *p*-value < 0.05 was considered statistically significant.

## 3. Results

### 3.1. Participant Characteristics

A total of 127 volunteers were assessed for eligibility, of whom 47 were excluded before randomization for not meeting the predefined inclusion or exclusion criteria. The remaining 80 participants were randomly assigned in a 1:1 ratio to receive either CA-HE50 (*n* = 40) or a placebo (*n* = 40).

During the 6-month intervention period, two participants in the CA-HE50 group and three in the placebo group were lost to follow-up due to loss of contact. As a result, 38 participants in the CA-HE50 group and 37 in the placebo group completed the study and were included in the PP analysis. All randomized participants were included in the ITT analysis.

Table 2 summarizes the baseline demographic and clinical characteristics of participants included in the PP analysis. There were no statistically significant differences between the CA-HE50 and placebo groups regarding sex distribution, age, height, body weight, body mass index (BMI), systolic and diastolic blood pressure, pulse rate, heart rate, respiratory rate, or body temperature at baseline (all *p* > 0.05), indicating that the groups were well balanced prior to the intervention.

### 3.2. Efficacy Outcomes

**Figure 4:**
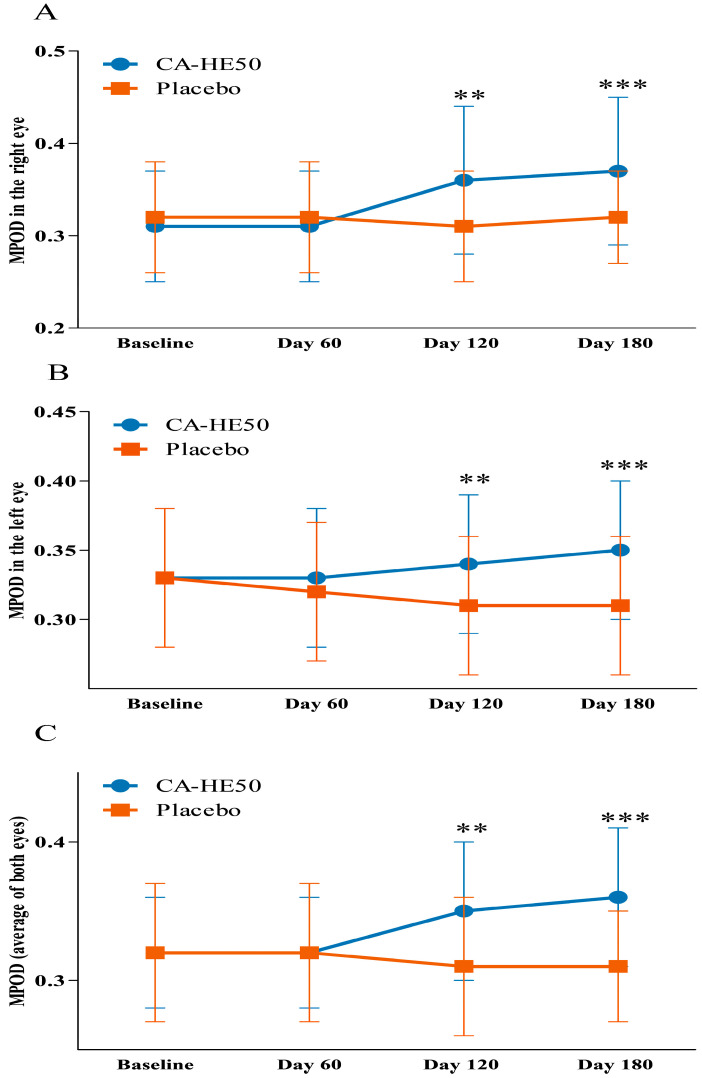
Changes in MPOD during the intervention period. MPOD values at baseline, Day 60, Day 120, and Day 180 are shown for the (**A**) right eye, (**B**) left eye, and (**C**) average of both eyes in the placebo and CA-HE50 groups. Values are presented as mean ± SD. An independent *t*-test was used to compare the groups at each time point. ** *p* < 0.01, *** *p* < 0.001 vs. placebo group.

**Figure 5:**
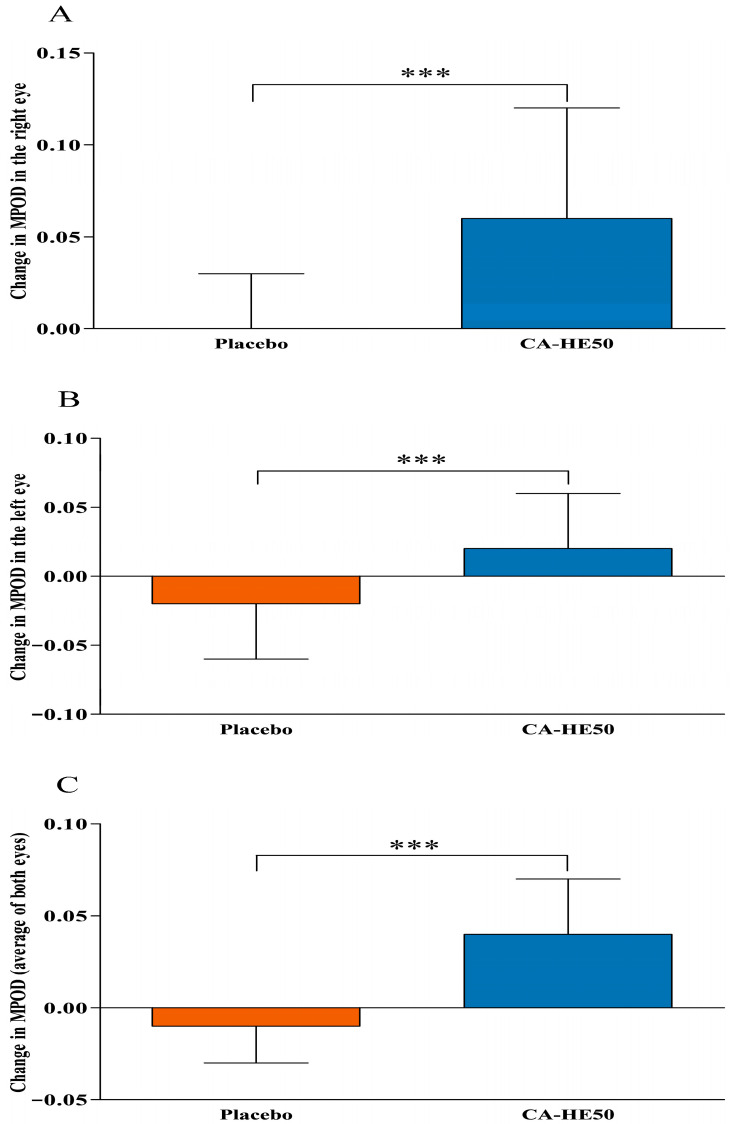
Changes in MPOD from baseline to Day 180. Changes in MPOD from baseline to Day 180 are shown for the (**A**) right eye, (**B**) left eye, and (**C**) average of both eyes in the placebo and CA-HE50 groups. Values are presented as mean ± SD. An independent *t*-test was used to assess the changes between the groups. *** *p* < 0.001 vs. placebo group.

Longitudinal changes in MPOD during the intervention period are summarized in Table 3 and illustrated in Figure 4. Changes from baseline to Day 180 (ΔMPOD) are presented in Figure 5.

At baseline, MPOD values for the right eye, left eye, and the average of both eyes were similar between the CA-HE50 and placebo groups (all *p* > 0.05), indicating that the two groups were well balanced in terms of macular pigment status at the start of the study.

In the right eye, the change in MPOD from baseline to Day 120 was significantly greater in the CA-HE50 group compared to the placebo group (0.05 ± 0.06 vs. −0.01 ± 0.03; *p* < 0.0001). Within-group analysis revealed a significant increase from baseline in the CA-HE50 group (*p* < 0.0001), while the change in the placebo group was not significant (*p* > 0.05). From baseline to Day 180, the increase in MPOD remained significantly greater in the CA-HE50 group compared to the placebo group (0.06 ± 0.06 vs. 0.00 ± 0.03; *p* < 0.0001). The within-group increase was significant in the CA-HE50 group (*p* < 0.0001), whereas no significant change was observed in the placebo group (*p* > 0.05).

In the left eye, the change in MPOD from baseline to Day 120 differed significantly between groups, favoring the CA-HE50 group (0.01 ± 0.03 vs. −0.02 ± 0.03; *p* = 0.0002). Within-group analysis revealed a significant increase in the CA-HE50 group (*p* = 0.0266) and a significant decrease in the placebo group (*p* = 0.0022). From baseline to Day 180, the change in MPOD was significantly greater in the CA-HE50 group than in the placebo group (0.02 ± 0.04 vs. −0.02 ± 0.04; *p* = 0.0001). Within-group comparisons indicated a significant increase in the CA-HE50 group (*p* = 0.0058) and a significant decrease in the placebo group (*p* = 0.0071).

For the average MPOD of both eyes, the change from baseline to Day 120 was significantly greater in the CA-HE50 group compared to the placebo group (0.03 ± 0.03 vs. −0.01 ± 0.02; *p* < 0.0001). Within-group analysis demonstrated a significant increase in the CA-HE50 group (*p* < 0.0001), while the change in the placebo group was also statistically significant (*p* = 0.0213). From baseline to Day 180, the change in average MPOD remained significantly greater in the CA-HE50 group than in the placebo group (0.04 ± 0.03 vs. −0.01 ± 0.02; *p* < 0.0001). Within-group analysis showed a significant increase in the CA-HE50 group (*p* < 0.0001), whereas the change in the placebo group was also significant (*p* = 0.0159).

In addition, analysis using a linear mixed-effects model demonstrated a significant group × time interaction for MPOD in the right eye (*p* < 0.0001), left eye (*p* = 0.0003), and the average of both eyes (*p* < 0.0001), indicating that longitudinal changes in MPOD differed significantly between the CA-HE50 and placebo groups during the intervention period.

Additional analyses using the ITT population (Table 4) showed results consistent with the PP analysis. The linear mixed-effects model also demonstrated significant group × time interactions for MPOD outcomes, further supporting the robustness of the efficacy findings.

**Figure 6:**
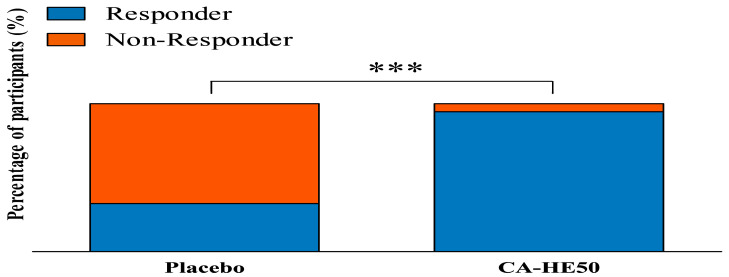
Proportion of responders and non-responders by group during the intervention period. Participants were classified as responders or non-responders based on predefined criteria for MPOD change. Values are presented as percentages. Fisher’s exact test was used to compare the groups. *** *p* < 0.001 vs. placebo group.

A responder analysis was conducted to further evaluate individual variability in MPOD response (Table 5, Figure 6). Responders were defined as participants who demonstrated an increase in MPOD from baseline to Day 180, reflecting a directional improvement in macular pigment during the intervention period. In the placebo group, 12 out of 37 participants (32.43%) were classified as responders, whereas 36 out of 38 participants (94.74%) in the CA-HE50 group met the responder criteria. In contrast, non-responders accounted for 25 participants (67.57%) in the placebo group and 2 participants (5.26%) in the CA-HE50 group. The distribution of responders and non-responders differed significantly between groups (*p* < 0.0001), indicating a substantially higher proportion of participants exhibiting MPOD improvement in the CA-HE50 group.

### 3.3. Safety Outcomes

Safety outcomes were assessed by examining AEs, vital signs, and clinical laboratory parameters.

During the 6-month intervention period, three participants in the CA-HE50 group and six in the placebo group reported AEs, with no significant between-group difference (*p* > 0.05). All reported AEs were mild to moderate in severity, which was deemed unrelated to the study product, and there were no serious adverse events. None of the participants discontinued the study due to AEs.

Table 6 summarizes vital sign measurements from baseline to Day 180. Throughout the intervention period, no clinically meaningful changes were observed in either group. Additionally, between-group comparisons showed no statistically significant differences in systolic blood pressure, diastolic blood pressure, pulse rate, heart rate, respiratory rate, or body temperature (*p* > 0.05 for all comparisons).

In addition, hematological parameters and blood biochemical markers remained within normal reference ranges throughout the study, showing no significant differences between the CA-HE50 and placebo groups (*p* > 0.05). These findings suggest that 6-month supplementation with CA-HE50 was well tolerated and presented no clinically relevant safety concerns.

## 4. Discussion

This randomized, double-blind, placebo-controlled clinical trial is the first to demonstrate that 6 months of supplementation with a standardized CA-HE50 significantly improves MPOD in adults aged 45 to 65 years with low baseline MPOD. Increases in MPOD were consistently observed in the right eye, left eye, and the average of both eyes, with between-group differences emerging at Day 120 and persisting through Day 180. These findings indicate that sustained nutritional intervention can meaningfully enhance macular antioxidant status even in non-diseased, middle-aged populations.

Participant selection in this study specifically targeted middle-aged adults with relatively low baseline MPOD instead of individuals with clinically diagnosed AMD. Previous research indicates that MPOD varies significantly among individuals during midlife and tends to decline with age, yet it remains responsive to nutritional interventions before advanced retinal pathology develops [35,36]. Consistent with this concept, dietary interventions, such as supplementation with zeaxanthin-rich goji berries (28 g/day for 90 days), have been shown to increase MPOD in healthy adults aged 45–65 years [37]. However, these methods often require a substantial intake of carotenoid-rich foods or high-dose supplements, which can hinder long-term adherence and practical application. In contrast, this study demonstrates that sustained supplementation with a low daily dose of a non-carotenoid botanical extract can significantly enhance MPOD in a similar middle-aged population. This finding suggests that altering the retinal antioxidant environment, rather than relying solely on direct pigment delivery, may offer a viable alternative strategy for improving macular pigment status during midlife.

In this study, long-term supplementation with CA-HE50 significantly improved MPOD, demonstrating its effectiveness in enhancing macular antioxidant status. While MPOD has typically been increased through the consumption of lutein- and zeaxanthin-rich foods or supplements, previous nutritional interventions have produced inconsistent results. For instance, supplementation with lutein and zeaxanthin for up to six months raised plasma carotenoid levels but did not affect MPOD [21]. Similarly, consuming lutein- and zeaxanthin-rich eggs resulted in little to no change in MPOD, despite increased serum carotenoid concentrations [38]. These varied responses are thought to be due to individual differences in carotenoid absorption, transport, and retinal uptake, as well as variations in the retinal oxidative environment. In this context, our findings reveal that CA-HE50 significantly enhanced MPOD even with lower intake levels and without direct carotenoid supplementation, underscoring its potential to influence macular antioxidant status beyond mere carotenoid availability.

Importantly, CA-HE50 supplementation led to a time-dependent improvement in MPOD, with significant increases observed at both 120 and 180 days, and a greater magnitude of change noted at the later time point. Since MPOD is regarded as a relatively stable retinal biomarker that reflects long-term carotenoid accumulation and oxidative balance, meaningful changes typically require sustained intervention over extended periods [39]. Previous studies have also indicated that longer intervention durations are more likely to yield measurable increases in MPOD [20]. Thus, the progressive improvement in MPOD seen in the CA-HE50 group highlights the importance of intervention duration and supports the idea of a cumulative effect achieved through modulation of the retinal oxidative environment, rather than through carotenoid availability alone.

The biological plausibility of these findings is supported by the pharmacological properties of *C. asiatica*, particularly its triterpenoid constituents: asiaticoside, madecassoside, asiatic acid, and madecassic acid. These bioactive triterpenoids represent the major characteristic components of *C. asiatica* extracts and are considered key contributors to their biological activity. These compounds exhibit antioxidant, anti-inflammatory, and cytoprotective properties through modulation of reactive oxygen species and cellular defense pathways [30,40]. Notably, asiaticoside has been shown to activate Nrf2 signaling, which results in the upregulation of antioxidant enzymes and enhances cellular resilience against chronic oxidative stress [32,41,42]. Given that oxidative stress plays an important role in retinal aging and macular degeneration, the antioxidant and cytoprotective effects of these triterpenoid compounds may help protect retinal pigment epithelial cells and maintain macular carotenoid stability. Such mechanisms may contribute to the preservation of macular pigment and provide a plausible explanation for the observed improvements in MPOD in the present study.

Preclinical studies utilizing the same standardized extract (CA-HE50) as in the current trial provide supportive mechanistic evidence for the clinical findings. In human retinal pigment epithelial cell models, CA-HE50 was shown to activate the Nrf2/HO-1 pathway, providing protection against oxidative and photo-oxidative stress [32]. In animal models of retinal degeneration, oral administration of CA-HE50 preserved retinal architecture and sustained the expression of proteins related to visual function, such as rhodopsin, thus mitigating functional decline in the retina [33]. These findings suggest that modulation of the retinal antioxidant environment may contribute to maintaining retinal cellular integrity. However, because MPOD primarily reflects retinal carotenoid concentration, the direct biological mechanism linking antioxidant modulation to changes in MPOD remains to be clarified in human studies.

The magnitude of MPOD increase observed in the present study (approximately 0.04–0.06 units) is comparable to the range reported in previous nutritional intervention studies. Clinical trials evaluating lutein and zeaxanthin supplementation have reported MPOD increases typically ranging from approximately 0.03 to 0.10 units, depending on supplementation dose, duration, and baseline MPOD status [14,15,21]. In this context, the increase observed with CA-HE50 supplementation falls within the range reported for established carotenoid-based interventions.

Although MPOD represents a structural biomarker rather than a direct functional outcome, accumulating evidence suggests that higher MPOD levels are associated with improvements in visual performance parameters, including contrast sensitivity, glare recovery, and visual processing speed [43,44]. These associations are thought to arise from the optical filtering and antioxidant properties of macular pigments within the retina. Therefore, while functional visual outcomes were not assessed in the present study, the observed increase in MPOD may have potential implications for visual performance and warrants further investigation in future studies incorporating functional visual assessments.

Moreover, responder analysis highlighted the clinical significance of these findings. The significantly higher proportion of responders in the CA-HE50 group indicates a relatively consistent effect among individuals. This contrasts with the considerable inter-individual variability often seen in carotenoid-based MPOD interventions [22,45,46], reinforcing the idea that modulating the retinal oxidative environment may lessen the impact of individual differences in carotenoid absorption and transport. It should be noted that responders in the present study were defined as participants who showed any increase in MPOD from baseline to Day 180. Although this approach captures directional improvement in macular pigment, small fluctuations due to measurement variability cannot be completely excluded. Previous studies have reported good test–retest repeatability for MPOD measurements obtained using heterochromatic flicker photometry [47]. Therefore, the markedly higher responder rate observed in the CA-HE50 group compared with the placebo group is unlikely to be explained solely by measurement variability. Nevertheless, future studies may benefit from applying predefined thresholds for clinically meaningful MPOD changes to further strengthen the interpretation of responder analyses.

CA-HE50 showed excellent tolerability throughout the 6-month supplementation period, with no clinically significant adverse events or negative changes in safety parameters. These results align with previous clinical studies of *C. asiatica* extracts, which reported improvements in liver function and cognitive performance without notable safety issues [48,49]. In contrast, some food-based MPOD interventions, such as egg consumption, have been linked to increases in serum cholesterol, raising concerns about potential long-term metabolic burdens in certain populations [50,51]. The lack of adverse lipid responses or safety signals in this study underscores the suitability of CA-HE50 as a well-tolerated and sustainable long-term nutritional strategy for enhancing MPOD.

This study has several limitations that should be acknowledged. First, participants were instructed to refrain from taking dietary supplements, including vitamin supplements and natural-product-based supplements, for 14 days prior to enrollment and throughout the intervention period. Accordingly, the use of carotenoid-containing dietary supplements was restricted during the trial. However, carotenoid intake derived from habitual dietary sources (e.g., lutein and zeaxanthin from foods) was not quantitatively assessed. Because MPOD can be influenced by dietary carotenoid intake, potential variations in carotenoid consumption through the regular diet during the 6-month intervention period cannot be entirely excluded. Nevertheless, the randomized, placebo-controlled design minimizes the likelihood of systematic dietary differences between the study groups. Second, the study did not include a positive control, such as carotenoid supplementation. The primary objective of this trial was to evaluate the efficacy of CA-HE50 compared with placebo under controlled conditions. Future studies directly comparing CA-HE50 with established carotenoid-based interventions may help further clarify its relative efficacy. Third, while MPOD was chosen as the primary outcome due to its established role as a non-invasive biomarker of macular antioxidant status, standard ophthalmologic examinations, including visual acuity assessments, were performed during study visits to monitor ocular status. However, additional functional visual parameters—such as contrast sensitivity and glare recovery—were not assessed. Including these outcomes in future research would provide a more comprehensive evaluation of the clinical significance of MPOD changes. Fourth, although the sample size was sufficient to detect statistically significant differences in MPOD between groups, the relatively modest sample size of this study may have limited the ability to conduct more detailed or exploratory subgroup analyses based on demographic or metabolic factors, which could be better addressed in future studies with a larger number of participants. Fifth, the responder analysis was based on any increase in MPOD from baseline, which may be sensitive to small measurement variability. Although MPOD measurements obtained using heterochromatic flicker photometry have demonstrated good test–retest repeatability in previous studies, minor fluctuations cannot be completely excluded. Future studies may benefit from applying predefined thresholds for clinically meaningful MPOD changes to further strengthen the interpretation of responder analyses. Finally, the study population comprised middle-aged adults with low baseline MPOD but without overt ocular disease, which may limit the generalizability of the findings to populations with advanced retinal pathology, and future studies including individuals with retinal diseases or more diverse clinical populations are warranted to further evaluate the broader applicability of CA-HE50.

Despite its limitations, this study boasts several significant strengths. Its randomized, double-blind, placebo-controlled design and six-month intervention period enhance internal validity and facilitate the assessment of sustained effects on MPOD, a biomarker typically resistant to short-term changes. Notably, this is the first clinical trial to demonstrate that a non-carotenoid botanical extract can significantly improve MPOD, thereby expanding our understanding beyond traditional carotenoid-based interventions. Additionally, the inclusion of mechanistic evidence from preclinical studies utilizing the same standardized extract bolsters the biological plausibility of the results. Furthermore, the favorable safety profile observed during long-term supplementation underscores the viability of CA-HE50 as a practical nutritional strategy for preserving macular health in midlife populations.

## 5. Conclusions

This randomized, double-blind, placebo-controlled clinical trial demonstrates that six months of supplementation with a standardized *Centella asiatica* extract (CA-HE50) significantly improves MPOD in middle-aged adults with low MPOD. Improvements were observed consistently in both eyes and were sustained throughout the intervention period. These results indicate that nutritional interventions can enhance macular antioxidant status during midlife, a time when retinal vulnerability starts to increase, even in the absence of obvious ocular disease. Additionally, the favorable safety profile associated with long-term supplementation reinforces the suitability of CA-HE50 as a nutritional strategy for maintaining macular health. Overall, this study provides clinical evidence for the potential role of CA-HE50 in improving MPOD and supports its value as a dietary strategy for promoting macular health in middle-aged individuals.

## Figures and Tables

**Table 1:** Composition of the study products administered during the intervention period.

Component	Placebo Group (%)	CA-HE50 Group (%)
*Centella asiatica* extract (CA-HE50)	0.00	33.33
Microcrystalline Cellulose	35.74	35.74
Milk sugar, lactose	25.00	25.00
Maltodextrin	33.33	0.00
Other excipients	5.93	5.93
Total	100.00	100.00

Values are expressed as a percentage of the total tablet weight (900 mg).

**Table 2:** Baseline characteristics of the participants.

	Placebo Group (*n* = 37)	CA-HE50 Group (*n* = 38)	Total (*n* = 75)	*p*-Value ^(1)^
Sex (M/F)	18/19	16/22	34/41	0.5693 ^(2)^
Age (years)	53.11 ± 6.30	54.63 ± 8.05	53.88 ± 7.23	0.3651
Height (cm)	162.41 ± 8.02	161.43 ± 8.97	161.91 ± 8.47	0.6228
Weight (kg)	66.08 ± 10.10	65.11 ± 18.85	65.59 ± 15.08	0.7801
BMI (kg/m^2^)	25.02 ± 3.10	24.00 ± 3.25	24.50 ± 3.20	0.1718
SBP (mmHg)	122.05 ± 5.06	122.63 ± 3.93	122.35 ± 4.50	0.5820
DBP (mmHg)	74.35 ± 5.75	73.66 ± 5.32	74.00 ± 5.51	0.5892
Pulse Rate (beats/min)	71.59 ± 3.10	72.21 ± 2.82	71.91 ± 2.96	0.3704
Heart rate (beats/min)	72.19 ± 3.36	73.05 ± 3.27	72.63 ± 3.32	0.2629
Respiratory rate (per/min)	19.16 ± 2.41	19.08 ± 2.27	19.12 ± 2.32	0.8781
Temperature (°C)	36.97 ± 0.06	36.91 ± 0.34	36.94 ± 0.25	0.3122

Values are presented as mean ± SD or N; ^(1)^ analyzed by an independent *t*-test between the groups; ^(2)^ analyzed by the chi-square test between the groups.

**Table 3:** Changes in MPOD in the right eye, left eye, and the average of both eyes during the intervention period (PP analysis).

		Placebo Group (*n* = 37)	CA-HE50 Group (*n* = 38)	*p*-Value ^(1)^	*d* ^(4)^
MPOD in the right eye	Baseline	0.32 ± 0.06	0.31 ± 0.06	0.3153	
	Day 60	0.32 ± 0.06	0.31 ± 0.06	0.4453	
	Change from baseline	0.00 ± 0.02	0.00 ± 0.01	0.1706	0.00
	*p*-value ^(2)^	0.2543	0.4401		
	Day 120	0.31 ± 0.06	0.36 ± 0.08	0.0079 **	
	Change from baseline	−0.01 ± 0.03	0.05 ± 0.06	<0.0001 ***	1.26
	*p*-value ^(2)^	0.1714	<0.0001		
	Day 180	0.32 ± 0.05	0.37 ± 0.08	0.0007 ***	
	Change from baseline	0.00 ± 0.03	0.06 ± 0.06	<0.0001 ***	1.26
	*p*-value ^(2)^	0.3782	<0.0001		
	Group × Time interaction ^(3)^			<0.0001 ***	
MPOD in the left eye	Baseline	0.33 ± 0.05	0.33 ± 0.05	0.7299	
	Day 60	0.32 ± 0.05	0.33 ± 0.05	0.5123	
	Change from baseline	−0.01 ± 0.01	0.00 ± 0.01	0.2006	1.00
	*p*-value ^(2)^	0.0008	0.1401		
	Day 120	0.31 ± 0.05	0.34 ± 0.05	0.0085 **	
	Change from baseline	−0.02 ± 0.03	0.01 ± 0.03	0.0002 ***	1.00
	*p*-value ^(2)^	0.0022	0.0266		
	Day 180	0.31 ± 0.05	0.35 ± 0.05	0.0006 ***	
	Change from baseline	−0.02 ± 0.04	0.02 ± 0.04	0.0001 ***	1.00
	*p*-value ^(2)^	0.0071	0.0058		
	Group × Time interaction ^(3)^			0.0003 ***	
MPOD (average of both eyes)	Baseline	0.32 ± 0.05	0.32 ± 0.04	0.6680	
	Day 60	0.32 ± 0.05	0.32 ± 0.04	0.8881	
	Change from baseline	0.00 ± 0.01	0.00 ± 0.01	0.1886	0.00
	*p*-value ^(2)^	0.6298	0.0919		
	Day 120	0.31 ± 0.05	0.35 ± 0.05	0.0025 **	
	Change from baseline	−0.01 ± 0.02	0.03 ± 0.03	<0.0001 ***	1.56
	*p*-value ^(2)^	0.0213	<0.0001		
	Day 180	0.31 ± 0.04	0.36 ± 0.05	<0.0001 ***	
	Change from baseline	−0.01 ± 0.02	0.04 ± 0.03	<0.0001 ***	1.96
	*p*-value ^(2)^	0.0159	<0.0001		
	Group × Time interaction ^(3)^			<0.0001 ***	

Values are presented as mean ± SD; ^(1)^ analyzed by an independent *t*-test between the groups; ^(2)^ analyzed by a paired *t*-test within each group; ^(3)^ analyzed using a linear mixed-effects model including group, time, and group × time interaction. ^(4)^ Effect sizes are presented as Cohen’s d. ** *p* < 0.01, *** *p* < 0.001 vs. placebo group.

**Table 4:** Changes in MPOD in the right eye, left eye, and the average of both eyes during the intervention period (ITT analysis).

		Placebo Group (*n* = 40)	CA-HE50 Group (*n* = 40)	*p*-Value ^(1)^	*d* ^(4)^
MPOD in the right eye	Baseline	0.32 ± 0.06	0.30 ± 0.06	0.2847	
	Day 60	0.32 ± 0.06	0.30 ± 0.06	0.3919	
	Change from baseline	0.00 ± 0.02	0.00 ± 0.01	0.1871	0.00
	*p*-value ^(2)^	0.2348	0.5703		
	Day 120	0.31 ± 0.06	0.36 ± 0.08	0.0079 **	
	Change from baseline	−0.01 ± 0.03	0.05 ± 0.06	<0.0001 ***	1.26
	*p*-value ^(2)^	0.1847	<0.0001		
	Day 180	0.32 ± 0.05	0.37 ± 0.08	0.0007 ***	
	Change from baseline	0.00 ± 0.03	0.06 ± 0.06	<0.0001 ***	1.26
	*p*-value ^(2)^	0.4048	<0.0001		
	Group × Time interaction ^(3)^			<0.0001 ***	
MPOD in the left eye	Baseline	0.33 ± 0.05	0.33 ± 0.05	0.7688	
	Day 60	0.32 ± 0.05	0.33 ± 0.05	0.5633	
	Change from baseline	−0.01 ± 0.01	0.00 ± 0.01	0.2305	1.00
	*p*-value ^(2)^	0.0004	0.0907		
	Day 120	0.31 ± 0.05	0.34 ± 0.05	0.0085 **	
	Change from baseline	−0.02 ± 0.03	0.01 ± 0.03	0.0002 ***	1.00
	*p*-value ^(2)^	0.0020	0.0258		
	Day 180	0.31 ± 0.05	0.35 ± 0.05	0.0006 ***	
	Change from baseline	−0.02 ± 0.04	0.02 ± 0.04	0.0001 ***	1.00
	*p*-value ^(2)^	0.0063	0.0055		
	Group × Time interaction ^(3)^			0.0003 ***	
MPOD (average of both eyes)	Baseline	0.32 ± 0.05	0.32 ± 0.04	0.6110	
	Day 60	0.32 ± 0.05	0.32 ± 0.04	0.8062	
	Change from baseline	0.00 ± 0.01	0.00 ± 0.01	0.2093	0.00
	*p*-value ^(2)^	0.5364	0.1857		
	Day 120	0.31 ± 0.05	0.35 ± 0.05	0.0025 **	
	Change from baseline	−0.01 ± 0.02	0.03 ± 0.03	<0.0001 ***	1.57
	*p*-value ^(2)^	0.0217	<0.0001		
	Day 180	0.31 ± 0.04	0.36 ± 0.05	<0.0001 ***	
	Change from baseline	−0.01 ± 0.02	0.04 ± 0.03	<0.0001 ***	1.96
	*p*-value ^(2)^	0.0162	<0.0001		
	Group × Time interaction ^(3)^			<0.0001 ***	

Values are presented as mean ± SD; ^(1)^ analyzed by an independent *t*-test between the groups; ^(2)^ analyzed by a paired *t*-test within each group; ^(3)^ analyzed using a linear mixed-effects model including group, time, and group × time interaction. ^(4)^ Effect sizes are presented as Cohen’s d. ** *p* < 0.01, *** *p* < 0.001 vs. placebo group.

**Table 5:** Comparison of responder and non-responder distributions between the groups during the intervention period.

	Placebo Group (*n* = 37)	CA-HE50 Group (*n* = 38)	*p*-Value ^(1)^
Responder	12 (32.43)	36 (94.74)	<0.0001 ***
Non-Responder	25 (67.57)	2 (5.26)	

Values are presented as *n* (%); ^(1)^ analyzed by Fisher’s exact test between the groups. *** *p* < 0.001 vs. placebo group.

**Table 6:** Changes in vital signs during the intervention period.

	Placebo Group (*n* = 37)				CA-HE50 Group (*n* = 38)				*p*-Value ^(1)^
	Baseline	Day 180	Change Value	*p*-Value ^(2)^	Baseline	Day 180	Change Value	*p*-Value ^(2)^	
SBP (mmHg)	122.05 ± 5.06	120.86 ± 2.29	−1.19 ± 5.44	0.1920	122.63 ± 3.93	120.47 ± 1.64	−2.16 ± 3.74	0.0010	0.3734
DBP (mmHg)	74.35 ± 5.75	73.27 ± 3.58	−1.08 ± 7.43	0.3346	73.66 ± 5.32	74.63 ± 4.63	0.97 ± 6.46	0.3590	0.2049
Pulse Rate (beats/min)	71.59 ± 3.10	71.05 ± 2.77	−0.54 ± 4.56	0.4311	72.21 ± 2.82	70.79 ± 2.56	−1.42 ± 4.04	0.0243	0.3787
Heart rate (beats/min)	72.19 ± 3.36	73.00 ± 2.46	0.81 ± 3.81	0.2042	73.05 ± 3.27	72.03 ± 2.73	−1.03 ± 4.26	0.1416	0.0533
Respiratory rate (per/min)	19.16 ± 2.41	20.14 ± 2.08	0.97 ± 2.11	0.0082	19.08 ± 2.27	20.63 ± 2.24	1.55 ± 2.84	0.0018	0.3209
Temperature (°C)	36.97 ± 0.06	36.95 ± 0.05	−0.02 ± 0.07	0.1769	36.91 ± 0.34	36.94 ± 0.06	0.03 ± 0.36	0.6228	0.4703

Values are presented as mean ± SD; ^(1)^ analyzed by an independent *t*-test between the groups; ^(2)^ analyzed by a paired *t*-test within each group.

## Data Availability

The datasets generated and analyzed during the current study are available from the corresponding author upon reasonable request. The data are not publicly available due to privacy and ethical restrictions.
